# Prediction of anatomically and biomechanically feasible precision grip posture of the human hand based on minimization of muscle effort

**DOI:** 10.1038/s41598-022-16962-1

**Published:** 2022-08-02

**Authors:** Takayuki Nakajima, Yuki Asami, Yui Endo, Mitsunori Tada, Naomichi Ogihara

**Affiliations:** 1 Department of Mechanical Engineering, Faculty of Science and Technology, Keio University, Yokohama, 223-8522 Japan; 2 Artificial Intelligence Research Center, National Institute of Advanced Industrial Science and Technology (AIST), Tokyo, 135-0064 Japan; 3 Department of Biological Sciences, Graduate School of Science, The University of Tokyo, 7-3-1, Hongo, Bunkyo-ku, Tokyo, 113-0033 Japan

**Keywords:** Biomedical engineering, Musculoskeletal system, Motor control

## Abstract

We developed a method to estimate a biomechanically feasible precision grip posture of the human hand for a given object based on a minimization of the muscle effort. The hand musculoskeletal model was constructed as a chain of 21 rigid links with 37 intrinsic and extrinsic muscles. To grasp an object, the static force and moment equilibrium condition of the object, force balance between the muscle and fingertip forces, and static frictional conditions must be satisfied. We calculated the hand posture, fingertip forces, and muscle activation signals for a given object to minimize the square sum of the muscle activations while satisfying the above kinetic constraints using an evolutionary optimization technique. To evaluate the estimated hand posture and fingertip forces, a wireless fingertip force-sensing device with two six-axis load cells was developed. When grasping the object, the fingertip forces and hand posture were experimentally measured to compare with the corresponding estimated values. The estimated hand postures and fingertip forces were in reasonable agreement to the corresponding measured data, indicating that the proposed hand posture estimation method based on the minimization of muscle effort is effective for the virtual ergonomic assessment of a handheld product.

## Introduction

Recently, ergonomic assessments of products or workspaces in virtual spaces using digital human models have gained considerable attention. Digital human modeling allows the simulation of mechanical interactions of the human body with products or workspaces, allowing virtual evaluation of the usability and safety of the products or workspaces, thereby reducing the cost and time required for product design and development. Therefore, these virtual techniques have been utilized to evaluate designs of products, such as automobile interiors and aircraft cockpits^1,2^.

Following this trend, attempts have been made to objectively evaluate the usability of handheld products based on simulated mechanical interactions of the human hand model with models of the products in a virtual space^3,4^. However, the human hand is a highly complex musculoskeletal system with many joints, muscles, and degrees of freedom; hence, there are a huge number of possible ways to grasp an object by hand. Although hand kinematics are somewhat specified from a huge number of possibilities, there are still a huge number of possible patterns to activate muscles to generate fingertip forces that satisfy the force and moment balance of grasping mechanics. Therefore, the challenge of computationally predicting realistic and anatomically natural grasping postures of the human hand for a given object in a virtual space persists. Subsequently, we address the question of how humans generate appropriate motor commands in muscles that lead to successful object grasping in an anatomically natural way.

Recent neurophysiological studies have suggested that the human central nervous system determines motor commands such that muscle effort, i.e., the sum of the squared muscle activations, is minimized^5–8^. If such a biologically feasible objective function can be specified, the grasping posture and muscle activation pattern necessary for a given object can be uniquely calculated by searching for the minimum point of the objective function that satisfies the force and moment equilibrium required for stable object grasping. This hand posture estimation method is potentially effective for virtual ergonomic assessment of a hand-held product.

Synthetic simulations of the human hand grasping an object have been conducted in the field of computer graphics for a realistic, natural animation of hand movements^9^. However, these simulations usually exploit a database of motion-captured hand poses to calculate the humanlike grasp posture for a given object. Moreover, measures to evaluate the quality of grasping, that is, the goodness of the grasping posture evaluated based on the stability or manipulability of the grasped object^10^, were often introduced to determine a unique solution for the problem^11^. However, these were not necessarily biologically plausible.

The aim of this study is to develop a method to estimate a geometrically and mechanically feasible grasping posture of the human hand for a given object based on a minimization of the neural effort. In this study, we attempt to incorporate the physiological aspects of human grasps into the synthesis of realistic, natural hand-object interactions in virtual environments. To evaluate whether the proposed methodology can replicate the kinematics and kinetics of the human hand grasping an object, we experimentally measured the fingertip forces and hand posture when grasping an object with the thumb and index finger using a fingertip-force sensing device and a motion-capture system, and compared them with corresponding estimated values.

## Materials and methods

### Hand model

For a realistic representation of the human hand, a computed tomography (CT) scan of a male hand (height: 1.69 m, weight: 63 kg, age: 50 years) was obtained using a CT scanner (Aquillion One, Canon Medical, Japan). The tube voltage and current were 120 kV and 400 mA, respectively. The pixel size and slice interval were 0.297 mm and 0.25 mm, respectively. Three-dimensional polygonal models of the hand surface and skeleton were then constructed.

**Figure 1:**
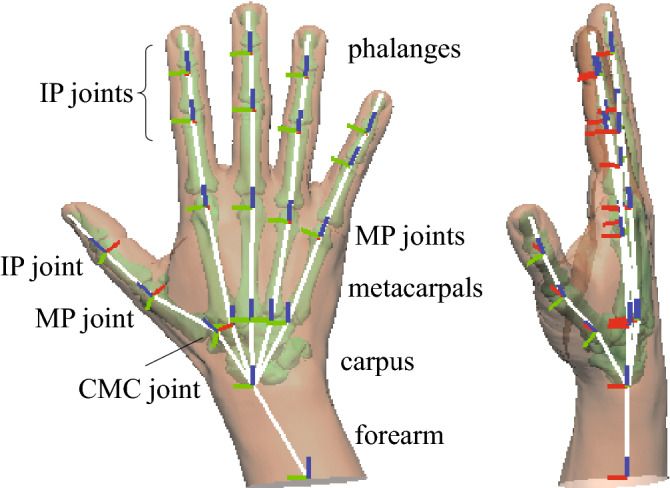
The hand musculoskeletal model.

The hand musculoskeletal model was modeled as a chain of rigid-body bone segments connected by revolute joints (Fig. 1). The carpal bones were treated as a single rigid body, disregarding inter-carpal mobility. Therefore, the hand was represented as a chain of 21 bone segments: the forearm, carpus, 1–5 metacarpals, and 14 phalanges. For each bone segment, a bone-fixed coordinate system was defined. The x-, y-, and z-axes roughly correspond to the dorso-palmar, medio-lateral, and proximo-distal directions, respectively. Each interphalangeal (IP) joint was represented by a hinge joint with one degree of freedom (DOF). The rotational axis and joint center were determined by approximating the proximal joint surface using a cylindrical surface by the least-squares method, and so was the first metacarpophalangeal (MP) joint. The second to fifth MP joints were each represented by a two-DOF revolute joint (flexion/extension and radial/ulnar rotation), the joint center of which was determined by approximating the proximal joint surface using a spherical surface. Each carpometacarpal (CMC) joint was represented by a gimbal joint. However, the second to fifth CMC joints were assumed to be immobile in this study. The joint center and rotational axes of the first CMC joints were determined by approximating the saddle-shaped joint surface using a hyperbolic paraboloid surface^12^.

A skin surface model of the hand was also generated from the CT data as a 3D polygonal mesh model. The surface naturally deforms as the joints move, based on a skin deformation algorithm^13^. The skin surface is necessary to represent the contact between the hand and grasped object. However, to reduce the computational cost, the surface of the hand was approximated by an aggregate of 65 spheres representing the hand surface, which was used to avoid the penetration of the hand into the object in the search for the grasping posture.

### Muscle model

As listed in Table 1, a total of 37 hand muscles were included in the hand model. Each muscle generates a force by receiving a muscle signal from a corresponding motor neuron, as follows:

1 $$F_{i} = F_{i}^{\max } a_{i} ,$$

**Table 1:** Muscle parameters of the hand musculoskeletal model.

Finger	No	Muscle	Abb	Max Force [N]	Moment arm [mm]				
					1CMC		1MP		1IP
					Flex-Ext	Add-Abd	Flex-Ext	Add-Abd	Flex-Ext
Thumb	1	Flexor pollicis longus	FPL	2.7	14.3	0.2	13.6	− 0.1	8.7
	2	Extensor pollicis longus	EPL	1.3	− 8.1	− 9.5	− 8.5	− 4.4	− 4.1
	3	Abductor pollicis longus	ABPL	3.1	− 7.1	10.5	0	0	0
	4	Extensor pollicis brevis	EPB	0.8	− 13	3.2	− 8.6	3.2	0
	5	Abductor pollicis brevis	ABPB	1.1	− 3.9	16.5	2.6	16.5	0
	6	Flexor pollicis brevis	FBP	1.3	13.4	10.5	8.8	10.5	0
	7	Opponens pollicis	OP	1.9	12.9	4.8	0	4.8	0
	8	Adductor pollicis transverse	APt	3	36.9	− 20.6	9.7	− 20.6	0
	9	Adductor pollicis oblique	APo	3	27	− 17	8.2	− 17	0

Finger	No	Muscle	Abb	Max Force [N]	Moment arm [mm]				
					2MP		2PIP	2DIP	
					Flex-Ext	Add-Abd	Flex-Ext	Flex-Ext	
Index	10	Flexor digitorum superficialis 2	FDS2	2	11.9	1.7	13.6	0	
	11	Flexor digitorum profundus 2	FDP2	2.7	10.2	0.4	− 8.5	4	
	12	Extensor digitorum communis 2	EDC2	1	− 9.4	0.7	0	− 1.6	
	13	Extensor indicis	EI	1	− 9.4	0.7	− 8.6	− 1.6	
	14	1st lumbrical	1LU	0.2	9.6	− 9.9	2.6	− 2	
	15	1st palmar interossei	1PI	1.3	6.4	8.8	8.8	− 2	
	16	1st dorsal interossei	1DI	3.2	4.4	− 9.6	0	0	

Finger	No	Muscle	Abb	Max Force [N]	Moment arm [mm]				
					3MP		3PIP	3DIP	
					Flex-Ext	Add-Abd	Flex-Ext	Flex-Ext	
Middle	17	Flexor digitorum superficialis 3	FDS3	3.4	11.5	0.6	5.3	0	
	18	Flexor digitorum profundus 3	FDS3	3.4	9.2	0.2	7.1	4.2	
	19	Extensor digitorum communis 3	EDC3	1.9	− 9.3	− 0.9	− 3.3	− 1.5	
	20	2nd lumbrical	2LU	0.2	10.5	8.2	− 2.8	− 1.9	
	21	2nd dorsal interossei	2DI	2.5	8	9.4	− 1.1	− 0.8	
	22	3rd dorsal interossei	3DI	2	3.4	− 7.6	− 2.6	− 1.8	

Finger	No	Muscle	Abb	Max Force [N]	Moment arm [mm]				
					4MP		4PIP	4DIP	
					Flex-Ext	Add-Abd	Flex-Ext	Flex-Ext	
Ring	23	Flexor digitorum superficialis 4	FDS4	2	9.9	− 1.2	5	0	
	24	Flexor digitorum profundus 4	FDP4	3	8.9	− 0.8	6.2	4.1	
	25	Extensor digitorum communis 4	EDC4	1.7	− 8.1	− 0.5	− 2.4	− 1.2	
	26	3rd lumbrical	3LU	0.1	6.6	− 7.5	− 2	− 1.5	
	27	2nd palmar interossei	2PI	1.2	3.1	− 7.6	− 2	− 1.5	
	28	4th dorsal interossei	4DI	1.7	4.7	7.1	− 1.2	− 0.9	

Finger	No	Muscle	Abb	Max Force [N]	Moment arm [mm]				
					5MP		5PIP	5DIP	
					Flex-Ext	Add-Abd	Flex-Ext	Flex-Ext	
Little	29	Flexor digitorum superficialis 5	FDS5	0.9	8.6	3.2	4.7	0	
	30	Flexor digitorum profundus 5	FDP5	2.8	8.5	4	5.9	3.2	
	31	Extensor digitorum communis 5	EDC5	0.9	− 4.9	0.9	− 2.6	− 1.3	
	32	Extensor digiti minimi	EDM	1	− 4.8	0.9	− 2.6	− 1.3	
	33	Abductor digiti minimi	ABDM	1.4	4.7	− 8	− 2	− 1.5	
	34	Flexor digiti minimi brevis	FDMB	0.4	7.9	0	0	0	
	35	4th lumbrical	4LU	2	6.3	7.2	− 2.2	− 1.7	
	36	3rd palmar interossei	3PI	1	2.1	7.7	− 2	− 1.5	
	37	Opponens digiti minimi	ODM	2	6	0	0	0	

Abb = abbreviation. CMC = Carpometacarpal joint, MP = Metacarpophalangeal joint, IP = Interphalangeal joint, PIP = Proximal IP joint, DIP = Distal IP joint.

where $F_{i}$ is the muscle force, $F_{i}^{\max }$ is the maximum muscle force, and *a*_*i*_ is the activation of the *i*th muscle (0 ≤ *a*_*i*_ ≤ 1). The maximum force of the muscles was determined as shown in Table 1 by referring to Li et al.^11^, who reported values based on the physiological cross-sectional areas of the hand muscles in the study by Brand and Hollister^14^ and specific muscle tension (35 N/cm^2^ ^15^). The pennation angle was not considered in the calculation because it is typically < 10° for hand muscles; thus, it does not significantly affect the force-generating capacity of the muscles.

The moment arms of the muscles were assumed to be constant, irrespective of the joint angle, and were determined with reference to An et al.^16^, Smutz et al.^17^, and Albrecht et al.^18^, as shown in Table 1. The joint torques generated by muscle activation ***a*** (vector of *a*_*i*_) can be written as

2 $${{\varvec{\uptau}}} = {\varvec{M}}^{T} {\varvec{F}}^{\max } {\varvec{a}},$$

where ${{\varvec{\uptau}}}$ is the joint torque vector, ${\varvec{M}}$ is the moment-arm matrix, and ${\varvec{F}}^{\max }$ is the diagonal matrix of $F_{i}^{\max }$.

### Grasping mechanics

**Figure 2:**
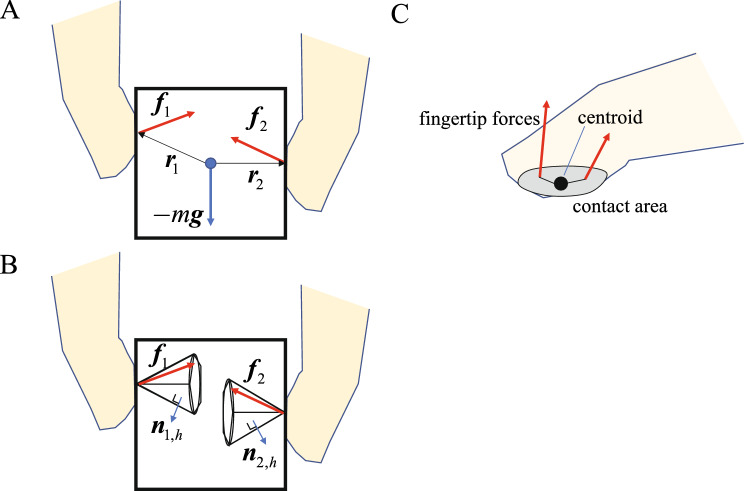
Grasping mechanics. (**A**) Forces applied to the object. (**B**) Friction cone approximated by a polygonal pyramid. The fingertip force vector must be positioned inside the friction cone. (**C**) Modeling of the contact of the distal phalanx. Two contact points 2 mm away from the centroid of the contact area were introduced to account for generation of a torsional moment during precision grip.

When the hand stably grasps an object, appropriate fingertip forces must be generated by the activation of muscles to achieve static equilibrium of forces and moments applied to the object (Fig. 2A). The force and moment equilibrium conditions of the object can be written as:

3 $$\left[ {\begin{array}{*{20}c} {\varvec{I}} & \cdots & {\varvec{I}} \\ {{\varvec{S}}({\varvec{r}}_{1} )} & \cdots & {{\varvec{S}}({\varvec{r}}_{n} )} \\ \end{array} } \right]\left[ {\begin{array}{*{20}c} {{\varvec{f}}_{1} } \\ \vdots \\ {{\varvec{f}}_{n} } \\ \end{array} } \right] = {\varvec{Gf}} = \left[ {\begin{array}{*{20}c} { - m{\varvec{g}}} \\ {\varvec{0}} \\ \end{array} } \right],$$

where ***I*** is a 3 × 3 identity matrix, ***S*** is a skew-symmetric matrix representing cross multiplication (${\varvec{S}}({\varvec{r}}_{k} ){\varvec{f}}_{k} = {\varvec{r}}_{k} \times {\varvec{f}}_{k}$), ${\varvec{r}}_{i}$ is the position vector connecting the center of mass (COM) of the object to the *k*th contact point where the *k*th fingertip force (${\varvec{f}}_{k}$) is applied, *m* is the mass of the object, and ***g*** is the gravitational acceleration. Matrix ***G*** is a 6 × 3*n* matrix referred to as a grasp matrix.

In addition, to achieve stable grasping of an object without slip, Coulomb’s law of static friction must be satisfied, i.e., tangential force must be smaller than the normal force multiplied by the friction coefficient (*µ*) of the contact surface. For stable contact without slip to occur, the fingertip force vector must be positioned inside the friction cone that determines the set of tangential and normal forces that can be applied to achieve contact without slip (Fig. 2B). Therefore, the apical angle of the frictional cone is given by $\theta = \tan^{ - 1} \mu$. If the friction cone is approximated by a polygonal pyramid (Fig. 2B), the condition necessary for contact without slip, i.e., for each fingertip force to always be positioned inside the friction cone, can be expressed as^11^:

4 $${\varvec{n}}_{k,h} {\varvec{f}}_{k} < {\varvec{0}},$$

where ${\varvec{n}}_{k,h}$ is the normal vector of the *h*th side surface of the *k*th frictional pyramid, corresponding to the *k*th contact (fingertip) force. In this study, each frictional cone was approximated using a twelve-sided pyramid.

In this study, we assumed that the contact between the object and each segment could be represented by a single contact point, the location of which was determined by the centroid of the contact area. Therefore, a torsional moment with respect to the normal at the contact point was not considered in the present study. However, the contact is actually a so-called soft contact allowing the finger to generate a torsional moment with respect to the normal at the contact point. To account for this, we introduced two contact points for the most distal end of each finger (distal phalanx), 2 mm away from the centroid of the contact area in a precision grip between the thumb and index finger (Fig. 2C).

### Mapping between fingertip forces and muscle activations

The muscles must be activated by the central nervous system to generate the fingertip forces necessary for successful grasping. Therefore, the relationship between fingertip forces and muscle activation should be established.

The relationship between the fingertip forces and joint torque ${{\varvec{\uptau}}}$ is given as

5 $${\varvec{J}}^{T} {\varvec{f}} = {{\varvec{\uptau}}},$$

where ***J*** is the 3*n* × 21 Jacobian matrix of the contact points with respect to the joint angles (${\varvec{J\dot{q} = \dot{c}}}$ where ***q*** is the 21 × 1 vector of joint angles and ***c*** is the 3*n* × 1 vector of the contact points represented in the global coordinate system) and ***f*** is the contact force vector ($${\varvec{f}} = \left[ {\begin{array}{*{20}c} {{\varvec{f}}_{1}^{{\text{T}}} } & {{\varvec{f}}_{2}^{{\text{T}}} } & { \cdot \cdot \cdot } & {{\varvec{f}}_{n}^{{\text{T}}} } \\ \end{array} } \right]^{{\text{T}}}$$). Therefore, the relationship between the muscle activation vector and fingertip force vector is

6 $${\varvec{J}}^{T} {\varvec{f}} - {\varvec{M}}^{T} {\varvec{F}}^{\max } {\varvec{a}} = {\varvec{0}}.$$

### Estimation of the grasping posture by minimizing muscle effort

The present study explored the kinematic posture of the hand model that minimizes the sum of squared muscle activations while satisfying grasping mechanics. The minimization problem can be formulated as

7 $$E = \sum\limits_{i = 1}^{37} {a_{i}^{2} } = {\varvec{a}}^{T} {\varvec{a}} \to \min .$$

Equation 7 is subject to the following linear equality and inequality constraints (Eqs. 3, 4, and 6 and range of muscle activation):

8 $$\begin{aligned} & {\varvec{Gf}} = \left[ {\begin{array}{*{20}c} { - m{\varvec{g}}} \\ {\varvec{0}} \\ \end{array} } \right] \\ & {\varvec{n}}_{k,h} {\varvec{f}}_{k} < {\varvec{0}} \\ & {\varvec{J}}^{T} {\varvec{f}} - {\varvec{M}}^{T} {\varvec{F}}^{\max } {\varvec{a}} = {\varvec{0}} \\ & 0 \le a_{i} \le 1. \\ \end{aligned}$$

This minimization problem can be reformulated as a quadratic programming problem of finding vector ***x*** ($${\varvec{x}} = \left[ {\begin{array}{*{20}c} {{\varvec{f}}_{1}^{{\text{T}}} } & {{\varvec{f}}_{2}^{{\text{T}}} } & { \cdot \cdot \cdot } & {{\varvec{f}}_{n}^{{\text{T}}} } & {a_{1} } & { \cdot \cdot \cdot } & {a_{37} } \\ \end{array} } \right]^{{\text{T}}}$$), which minimizes the following quadratic objective function that is identical to Eq. (7):

9 $$E = \frac{1}{2}{\varvec{x}}^{{\text{T}}} \left[ {\begin{array}{*{20}c} {{\varvec{0}}_{3k,3k} } & {{\varvec{0}}_{3k,37} } \\ {{\varvec{0}}_{37,3k} } & {{\varvec{I}}_{37,37} } \\ \end{array} } \right]{\varvec{x}} \to {\text{min}}$$

subject to

10 $$\left[ {\begin{array}{*{20}c} {\varvec{G}} & {{\varvec{0}}_{6,37} } \\ {{\varvec{J}}^{{\text{T}}} } & { - {\varvec{M}}^{{\text{T}}} {\varvec{F}}^{{{\text{max}}}} } \\ \end{array} } \right]{\varvec{x}} = \left[ {\begin{array}{*{20}c} { - m{\varvec{g}}} \\ {{\varvec{0}}_{21} } \\ \end{array} } \right],$$

11 $$\left[ {\begin{array}{*{20}c} {{\varvec{N}}_{12n,3n} } & {{\varvec{0}}_{12n,37} } \\ {{\varvec{0}}_{37,3n} } & {{\varvec{I}}_{37,37} } \\ {{\varvec{0}}_{37,3n} } & { - {\varvec{I}}_{37,37} } \\ \end{array} } \right]{\varvec{x}} \le \left[ {\begin{array}{*{20}c} {{\varvec{0}}_{12n} } \\ {{\varvec{1}}_{37} } \\ {{\varvec{0}}_{37} } \\ \end{array} } \right],$$

where ***N*** is a matrix of the normal vectors of the side surfaces of the frictional pyramids, defined as:

12 $${\varvec{N = }}\left[ {\begin{array}{*{20}c} {{\varvec{n}}_{1} } & {{\varvec{0}}_{12,3} } & \cdots & {{\varvec{0}}_{12,3} } \\ {{\varvec{0}}_{12,3} } & {{\varvec{n}}_{2} } & \cdots & {{\varvec{0}}_{12,3} } \\ \vdots & \vdots & \ddots & \vdots \\ {{\varvec{0}}_{12,3} } & {{\varvec{0}}_{12,3} } & \cdots & {{\varvec{n}}_{n} } \\ \end{array} } \right],\;\;{\varvec{n}}_{k} = \left[ {\begin{array}{*{20}c} {{\varvec{n}}_{k1}^{{\text{T}}} } \\ \vdots \\ {{\varvec{n}}_{k12}^{{\text{T}}} } \\ \end{array} } \right].$$

### Inverse kinematics of the hand

The fingertips must be in contact with the surface of the grasped object. Therefore, to determine the grasping posture that minimizes muscle effort, we searched for the positions of the fingertips on the surface of the object, and the corresponding hand posture was calculated by solving the inverse kinematics. Specifically, the position and orientation vectors of the carpal segment (***p***, ***e***) and joint angle vector (***q***) were calculated for the given target fingertip positions to minimize the objective function (*L*) using a quasi-Newtonian method.

13 $$\begin{aligned} L &= w_{1} \sum\limits_{l = 1}^{5} {\left| {{\varvec{d}}_{l} ({\varvec{p}},{\varvec{q}},{\varvec{e}}) - {\varvec{d}}_{l}^{0} } \right|^{2} } + w_{2} \sum\limits_{m = 1}^{65} {s_{m}^{2} } + w_{3} \left| {{\varvec{p}} - {\varvec{p}}^{0} } \right|^{2} \hfill \\ & \quad+ w_{4} \left| {{\varvec{e}} - {\varvec{e}}^{0} } \right|^{2} + w_{5} \left| {{\varvec{q}} - {\varvec{q}}^{0} } \right|^{2} \to \min , \hfill \\ \end{aligned}$$

where ${\varvec{d}}_{l}^{{}}$ is the position of the *l*th fingertip ($l = 1\sim 5$), represented as a function of ***p***, ***e***, and ***q***; ${\varvec{d}}_{l}^{0}$ is the corresponding target position; $s_{m}$ is the square of the penetration depth of the hand surface represented by the 65 spheres and grasped object; ${\varvec{p}}^{0}$ and ${\varvec{e}}^{0}$ are the initial position and orientation of the carpal segment, respectively; ${\varvec{q}}^{0}$ are the initial (anatomically natural) joint angles of the hand; and $w_{1} \sim w_{5}$ are the weighting coefficients. Therefore, the hand posture was calculated such that the fingertips were in contact with the object at the target positions, thereby ensuring that the hand surface did not penetrate the object while minimizing deviations from the neutral joint angles of the hand.

### Computational flow

Given the initial guess of the fingertip positions and the carpal segment position and orientation, hand kinematics were calculated based on Eq. (13). Subsequently, the fingertip forces and muscle activations were calculated by minimizing the quadratic function in Eq. (9) under the constraints of Eqs. (10–12), and the value of objective function *E* was calculated. If no solution satisfied the constraints, a sufficiently large value (sum of square error between the current and initial hand postures) was assigned to *E*. The fingertip positions were updated, and the above calculations were repeated until objective function *E* converged to a minimum point. We used the covariance matrix adaptation evolution strategy (CMA-ES) algorithm^19^ for this optimization process.

However, it must be noted that human fingertip force control includes a safety margin that is higher than the actual fingertip forces necessary to stably grasp an object against unexpected perturbations^20,21^. In the present study, to account for this, we assumed that the central nervous system estimated the friction coefficient (*µ*) of the object surface to be half of the actual value (if the value of *µ* is 0.8, it is estimated to be 0.4 in the central nervous system for grasping).

### Experimental validation of the model

**Figure 3:**
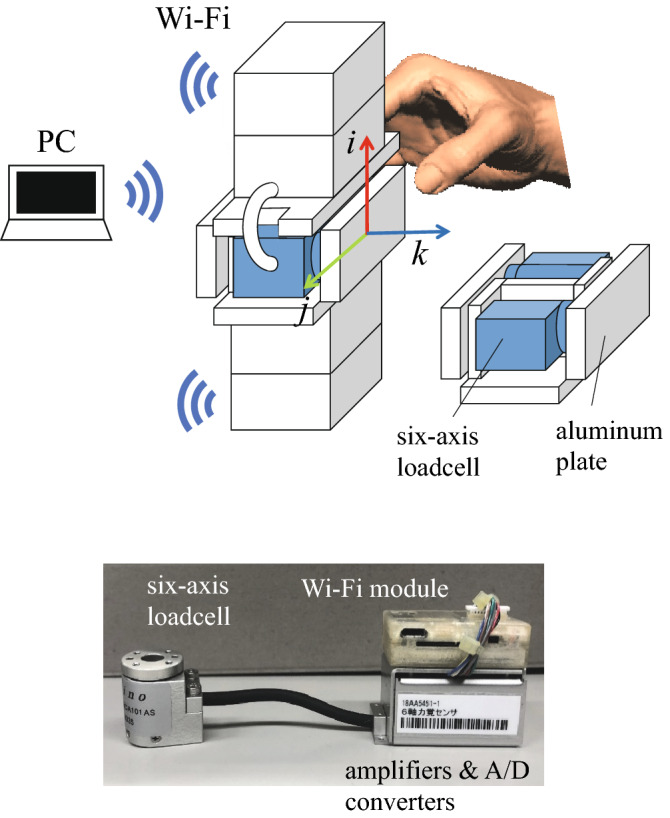
Custom-made wireless fingertip force-sensing device. The device consisted of two six-axis load cells, amplifiers, A/D converters, and Wi-Fi modules with batteries. An aluminum plate (20 mm × 49 mm) was attached to each load cell so that the forces applied by the thumb and index fingers could be measured independently. The amplifiers, A/D converters, and Wi-Fi modules with batteries were attached to the top and bottom of the load cell housing. The vectors *i,j,k* represent the sensor-fixed local coordinate system.

To evaluate whether the proposed methodology can replicate the kinematics and kinetics of a human hand grasping an object, it is necessary to experimentally quantify the three-dimensional fingertip forces and hand posture simultaneously when grasping an object. For this purpose, we developed a custom-made wireless fingertip force-sensing device. The device consisted of two six-axis load cells (CFS018CA101AS, Leptrino, Komoro, Japan), amplifiers, A/D converters, and Wi-Fi modules with batteries. The load cells were arranged in opposite directions and an aluminum plate (20 mm × 49 mm) was attached to each load cell (Fig. 3) so that the forces applied by the thumb and index fingers could be measured independently. The amplifiers, A/D converters, and Wi-Fi modules with batteries were attached to the top and bottom of the load cell housing. The device had a weight of 198.4 g. The device is unique in that the electric power is supplied from the batteries in the device, and the data is transmitted to a tablet PC via Wi-Fi communication. Hence, the device has no physical contact with the outside, allowing for precise physiological investigations of how the central nervous system achieves the force and moment balance of the grasped object.

**Figure 4:**
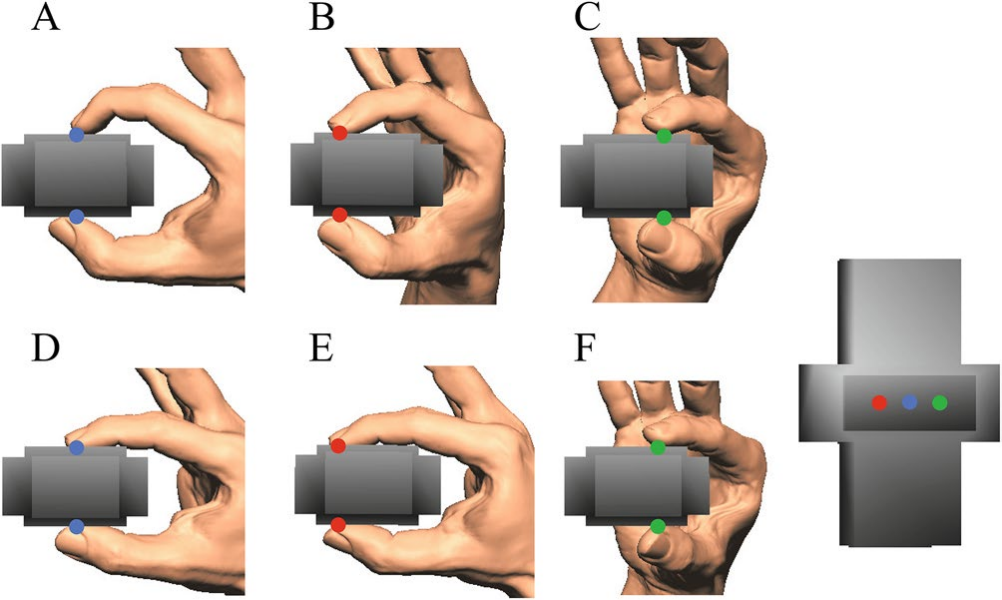
Six measured conditions. We asked the participant to pinch the center, far side, and near side of the device with fingertips (**A**, **B**, and **C**, respectively) and finger pulps (**D**, **E**, and **F**, respectively), and the forces applied to the device and the centers of pressure were measured.

**Figure 5:**
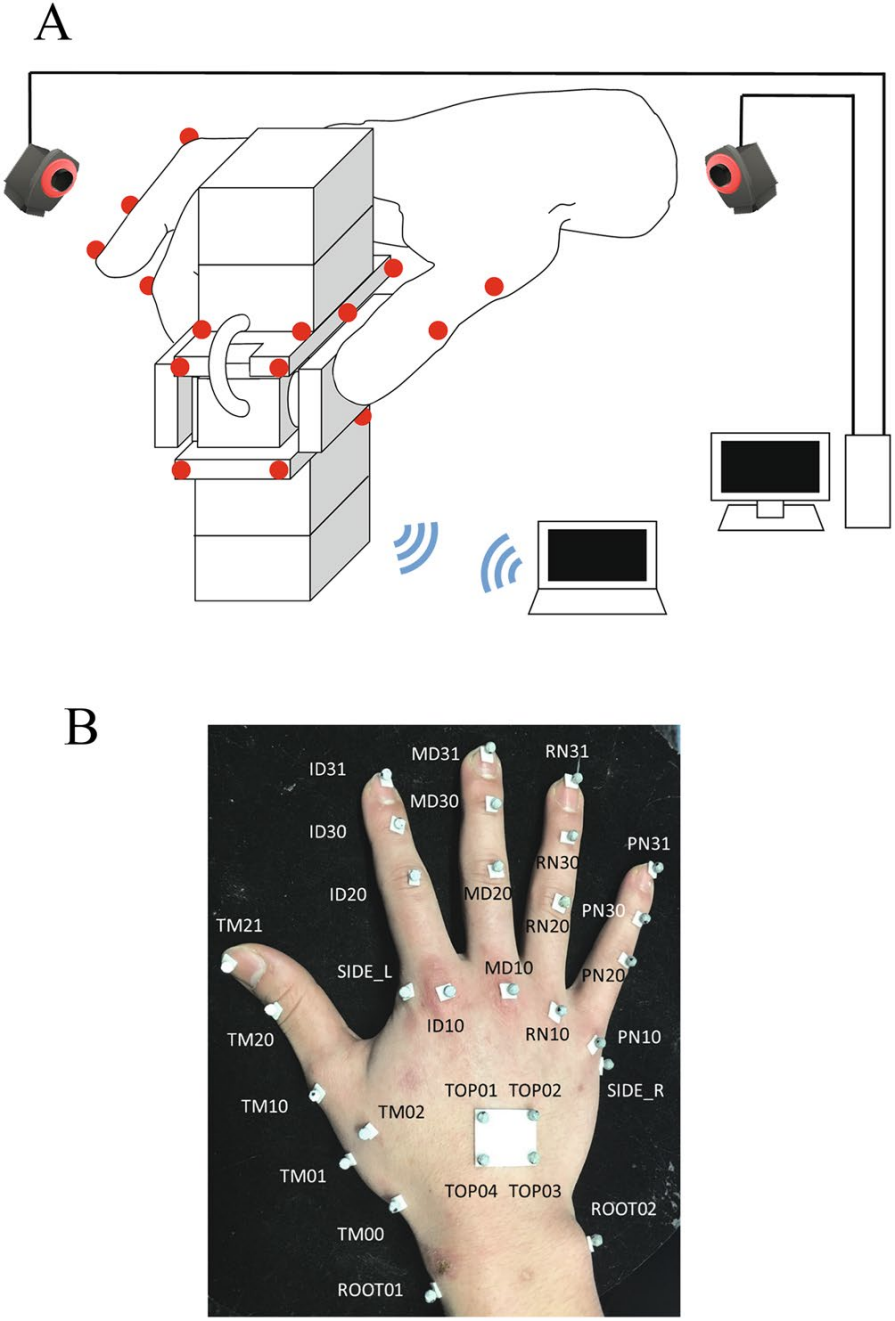
Measurement of hand kinematics using a motion capture system (**A**) and placement of reflective markers on the dorsal surface of the hand (**B**).

In the present study, we asked one male participant (178 cm, 63 kg, right-handed), whose hand was approximately equal in size to the hand model, to pinch, lift, and hold the fingertip force-sensing device in the air using the thumb and index finger under different conditions. Specifically, we asked the participant to pinch the center, far side, and near side of the device (Fig. 4) with fingertips (Conditions A, B, and C, respectively) and finger pulps (Conditions D, E, and F, respectively), and the forces applied to the device and the centers of pressure were measured. The participant was instructed to pinch the device as naturally as possible, and no instructions regarding muscle effort and fingertip forces were provided. Two types of precision grip were compared to check if the proposed method can correctly predict that precision grip using fingertips requires larger muscle effort than that using finger pulps. The hand kinematics were recorded using a motion capture system consisting of 17 cameras (OptiTrack; NaturalPoint, Corvallis, OR, USA) (Fig. 5A). A total of 30 reflective markers (4 mm diameter) were attached to the dorsal surfaces of five fingertips; five distal, four intermediate, and five proximal phalangeal joints; five metacarpophalangeal joints; first carpometacarpal joint; four points on the dorsal surface of the carpal bones; radial side of the second metacarpal head; ulnar side of the fifth metacarpal head; ulnar and radial styroid processes; and dorsoradial and dorsoulnar surfaces of the midpoint of the first metacarpal to capture the hand kinematics during the precision grip task (Fig. 5B). We additionally captured the positions of the 12 markers placed on the grasped device (Fig. 5A) to quantify the position and orientation of the grasped object. Measurement was conducted once for each condition confirming that grasping of the device was quite stereotyped at rehearsal. The experiment took about 90 min. Informed consent was obtained from the participant. This study was approved by the Committee on Ergonomic Experiments, AIST (HF2020-618). All methods were performed in accordance with the relevant guidelines and regulations.

We replicated the measured hand pose and object in a virtual space using the motion-captured marker positions to minimize the sum of distances between each motion-captured marker and corresponding markers on the hand model. It must be noted, however, that the calculated hand model could penetrate the object or was not in contact with the object because the hand model was not identical to the hand on the actual subject, and there were tiny discrepancies between the position of the markers on the actual hand and those on the model. Therefore, we adjusted the hand posture in the vicinity of the object to minimize the square sum of muscle activations while satisfying the kinematic (i.e., the thumb and index finger were on the aluminum plates of the object) and kinetic constraints (Eqs. (10)–(12)) that must be satisfied for object grasping. By comparing the magnitudes and directions of the measured and estimated fingertip force vectors, we evaluated how well the proposed method could predict grasping kinetics. The accuracy and precision of each force component and moment were evaluated by calculating the mean and standard deviation of the differences between the measured and estimated values.

In addition, we asked the participant to freely pinch the device in a self-selected manner and evaluated whether the proposed method could predict the actual fingertip positions, hand pose, and fingertip forces for a given object based on the present iterative method. In the CMA-ES optimization, the step size was set sufficiently large such that the entire search space (aluminum plate surfaces) was sampled to avoid getting trapped in a local minimum. We solved this optimization problem by using two different sets of initial values (Case I and II, far end and near end of the aluminum plates, respectively) to confirm the convergence of the solution.

## Results

**Figure 6:**
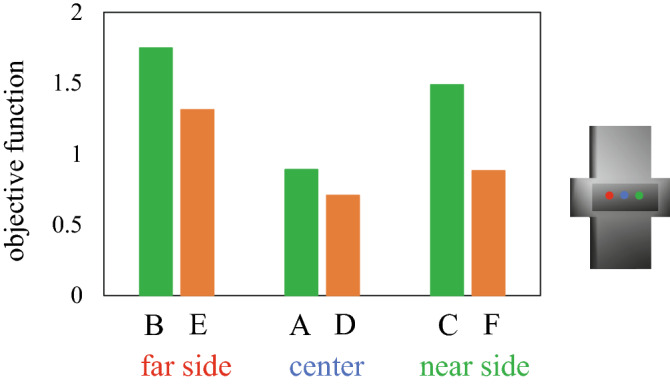
Comparison of the objective functions when the participant pinched the center, far side, and near side of the device with fingertips (**A**, **B**, and **C**, respectively) and finger pulps (**D**, **E**, and **F**, respectively). The value was smaller if the object was held with the finger pulps (orange) rather than the fingertips (green).

Table 2 compares the estimated fingertip forces and moments of the thumb and index finger with the corresponding measured values under the six conditions. The forces and moments are represented by an object-fixed local coordinate system. The estimated fingertip forces generally agreed with the corresponding measured data. The values of the objective function after the optimization are compared in Fig. 6. The value of the objective function tended to be smaller when the object was held at the center of each plate (Conditions A and D). The value was smaller if the object was held with the finger pulps rather than the fingertips.

**Table 2:** Comparisons of measured and simulated forces and moments of six grasping conditions.

	Thumb			Index			Thumb	Index
	F_i_ [N]	F_j_ [N]	F_k_ [N]	F_i_ [N]	F_j_ [N]	F_k_ [N]	M_k_ [Nm]	M_k_ [Nm]
**Fingertip**								
*A. center*								
measured	0.90	− 0.32	− 2.62	1.05	0.18	2.64	− 0.0001	− 0.0011
simulated	0.90	− 0.63	− 3.02	1.04	0.54	3.03	− 0.0019	− 0.0023
Δ	0.00	− 0.32	− 0.40	− 0.01	0.35	0.39	− 0.0018	− 0.0013
*B. far side*								
measured	0.34	− 0.26	− 8.11	0.68	− 1.30	8.08	0.0034	− 0.0016
simulated	0.55	− 0.93	− 7.35	0.71	− 0.55	7.42	− 0.0043	− 0.0028
Δ	0.21	− 0.68	0.77	0.03	0.76	− 0.66	− 0.0078	− 0.0012
*C. near side*								
measured	0.16	− 0.46	− 5.03	0.84	− 1.09	4.94	− 0.0005	− 0.0066
simulated	0.34	− 0.93	− 4.85	0.52	− 0.81	4.78	− 0.0084	− 0.0069
Δ	0.19	− 0.47	0.18	− 0.32	0.28	− 0.17	− 0.0079	− 0.0003
**Finger pulp**								
*D. center*								
measured	0.84	− 0.37	− 3.70	1.09	0.32	3.79	− 0.0033	− 0.0069
simulated	1.30	0.32	− 3.60	0.95	− 0.19	3.61	− 0.0015	− 0.0052
Δ	0.45	0.69	0.10	− 0.13	− 0.51	− 0.18	0.0018	0.0017
*E. far side*								
measured	0.66	− 0.62	− 4.06	1.27	0.63	4.09	− 0.0063	0.0029
simulated	1.31	0.36	− 3.49	0.64	− 0.42	3.51	0.0005	0.0037
Δ	0.65	0.98	0.58	− 0.64	− 1.05	− 0.58	0.0068	0.0008
*F. near side*								
measured	0.13	− 0.66	− 3.31	0.78	− 0.92	3.44	− 0.0021	− 0.0077
simulated	0.98	− 0.94	− 3.92	− 0.19	− 0.84	3.90	0.0007	− 0.0110
Δ	0.84	− 0.28	− 0.61	− 0.97	0.09	0.45	0.0027	− 0.0033
Accuracy	0.39	− 0.01	0.10	− 0.34	− 0.01	− 0.12	− 0.0010	− 0.0006
Precision	0.32	0.68	0.54	0.39	0.65	0.47	0.0059	0.0018

F_i_, F_j_, and F_k_ are the load (vertical), transverse and grip (normal) forces, respectively, represented in the object coordinate system.

M_k_ is the torsional moment at the center of pressure around a normal to the contact surface.

**Figure 7:**
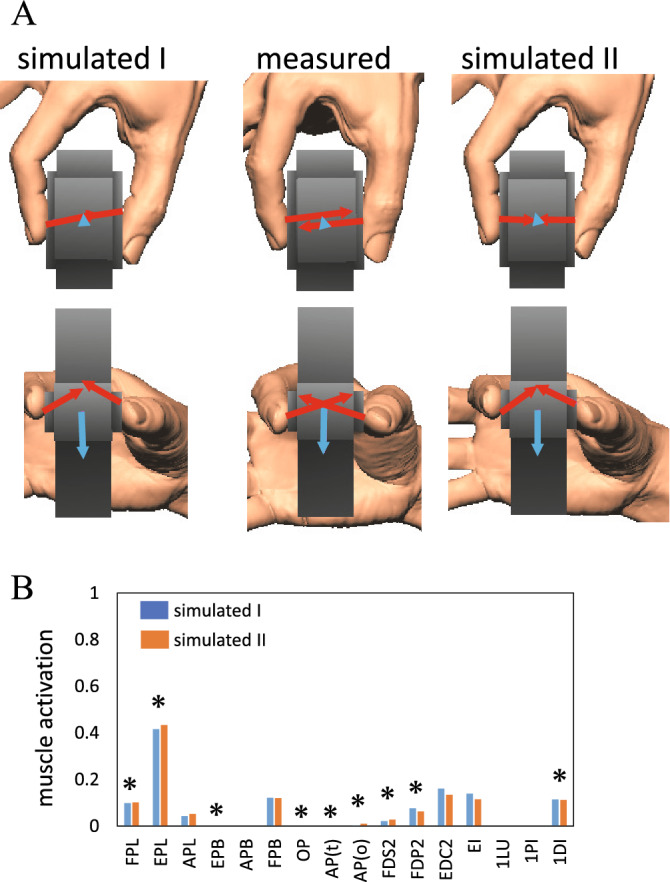
Comparisons of the measured fingertip forces of the free pinch with those estimated based on the present method from the two sets of initial values (I and II) (**A**). The estimated muscle activations are also presented (**B**). Asterisks indicated muscles that are reportedly active during precision grip^25–27^.

The measured fingertip forces and moments of the free pinch were compared with those estimated based on the present method, and the comparison is presented in Table 3 and Fig. 7. The values of the objective function obtained from an optimization that started from the two sets of initial values (I and II) were almost identical (= 0.264), indicating that quasi-global optimal solutions were obtained using the CMA-ES. The estimated fingertip forces were generally comparable to the corresponding measured data; however, the normal components of the estimated fingertip forces (grip forces) in the simulation were substantially smaller than those in the measurement. Furthermore, the value of the objective function for the measured grasping posture was 0.398, indicating that the estimated grasping kinetics in this study were better in minimizing the muscle effort than those that were measured. The differences in the joint angles were − 9.5 ± 20.3 degrees for the case I and − 4.7 ± 14.3 degrees and for case II (corresponding to 12.4% and 8.5% of joint range of motion^22^, respectively), indicating that the hand kinematics for a given object were predicted with reasonable accuracy considering the fact that the hand model was not identical to the measured hand. The estimated muscle activations are presented in Fig. 7B. The present simulation predicted that FPL, EPL, APL, FPB, FDS2, FDP2, EDC2, EI, and 1DI were activated during the free pinching of the object.

**Table 3:** Comparisons of measured and simulated forces and moments of free pinch.

	Thumb			Index			Thumb	Index
	F_i_ [N]	F_j_ [N]	F_k_ [N]	F_i_ [N]	F_j_ [N]	F_k_ [N]	M_k_ [Nm]	M_k_ [Nm]
**Measured**	0.98	0.52	− 3.03	0.97	− 0.62	3.11	− 0.0052	− 0.0010
**Simulated**								
I	0.93	0.32	− 1.69	1.01	− 0.32	1.69	0.0000	− 0.0002
II	0.78	0.02	− 1.68	1.17	− 0.02	1.68	0.0001	− 0.0002

## Discussion

The present study proposes a physiologically based method to predict the precision grip posture of the human hand. Specifically, the minimization of muscle effort, i.e., the sum of squared muscle activations, was used as an objective function to search for the grip posture. Overall, the proposed method reasonably estimates the kinematics and kinetics of hand grasping for a given object. Therefore, the proposed method can be used to virtually evaluate how a change in the design and dimensions of a product such as a camera alters its usability, without conducting ergonomic sensory evaluation by creating physical mockups of the product with different designs or dimensions, thereby reducing the cost and time required for product design and development.

It should be noted, however, that the predicted fingertip forces and the objective function of the predicted grip posture were slightly lower than that of the measured grip posture. This possibly indicates that the grip kinematics and kinetics for a given object may not be rigorously determined by the exact global optimal solution but are loosely determined within a range of quasi-optimal solutions in the vicinity of the global optimal solution. This is reasonable because a small deviation from the true optimal solution is not significantly different from the optimal solution, and no critical differences exist in of the mechanics and energetics of grasping. This is probably one of the reasons why there is a certain range of variability in the grasping kinematics and kinetics for a certain object. Therefore, the present study indicated that the proposed method for predicting the grasping posture of the human hand based on a minimization of the muscle load may be effective for the virtual ergonomic assessment of a hand-held product.

The present study predicted that the objective function (muscle effort) would be smaller when the object was held at the center than when it was held at the far and near sides (Fig. 6). This is because the COM of the object was located at the center of the object. When the object was pinched at the center by the thumb and index finger, the torsional moments due to gravitational force were only minimally applied to the fingertips because of the small moment arms. Conversely, if the object was pinched away from the COM, relatively large torsional moments that needed to be balanced by larger muscle activations were generated at the fingertips. In addition, the objective function was predicted to be larger when the object was grasped only by the fingertips than when it was grasped by the finger pulp. This was because larger joint torques, hence muscle forces, were necessary to generate a force at the distal end of the finger rather than somewhere relatively proximal. Therefore, the estimated values of the objective function for the six conditions were reasonable for the mechanics of object grasping.

Another unique contribution of the present study is that we attempted to validate the proposed estimation method by measuring the actual forces applied to a grasped object using a custom-made wireless fingertip force-sensing device. To clarify the kinetics of a human precision grip, previous studies have attempted to measure the fingertip forces applied to an object during the precision grip^23,24^. However, in these previous studies, the grasped object was connected to an electronic wire, making comparisons between the predicted and measured fingertip forces difficult because an unknown external force could be applied to the object in addition to the fingertip forces due to the wire. To the best of our knowledge, this is the first study attempting to construct an experimental setup allowing complete investigation of the kinetics of a precision grip, as well as experimentally validating the simulation of object grasping.

In the present study, we did not record electromyography (EMG) signals of the hand muscles for the evaluation of the predicted muscle activation because of the invasive nature of EMG recordings using wire electrodes, and the inability to separate individual hand muscle signals using surface electrodes. Nevertheless, although direct comparisons were not possible, we compared the muscle activations in Fig. 7B with those in previously published EMG data obtained during a human precision grip^25–27^. The predicted activations of FPL, EPL, FDS2, FDP2, and 1DI were consistent with those reported previously. However, the present study predicted that APL, FPB, EDC2, and EI were active, although they were reportedly inactive, and OP and AP were inactive, although they were reportedly active during precision grip. Therefore, the predicted muscle activations were not in perfect agreement with those in the previous reports. This discrepancy could be due to the differences between the kinematic and kinetic conditions of the precision grip of the previous and present studies. For a more rigorous validation of the proposed estimation method, in vivo simultaneous recordings of the fingertip forces and EMGs of the hand muscles during object grasping are necessary and should be addressed in future studies.

The discrepancies between the predicted and measured forces, moments, and muscle activation could also be due to errors associated with modeling the human hand. In this study, the moment arms of the muscles were assumed to be constant irrespective of changes in joint angles, but this simplification was not valid and might have affected the estimation results. For more precise modeling, each muscle path should be defined as a series of points connected by line segments (e.g., Mirakhorlo et al.^28^; Saito et al.^29^), and the dorsal aponeurosis of the finger should be modeled as a web-like structure^30,31^. Also, the present model did not incorporate passive elastic elements around the joints and muscle properties, such as force–length and force–velocity relationships (e.g., Zajac^15^). Further improvements in the musculoskeletal model might also be necessary to improve the accuracy of the predicted kinematics and kinetics of the hand grasping an object. In addition, this study considered grasping an object using only two fingers. We believe that an extension of the proposed method to entire-hand grasping is easy, but this should be confirmed. Furthermore, the present posture estimation method is based on inverse dynamics, but forward dynamics can also be employed to predict the hand posture for a given object^32,33^. This should be investigated further in future studies. Finally, the present study compared the estimated kinematic and kinetics of the hand model with the corresponding experimental data of the participant whose hand was equal in size to the hand model. However, for more precise validation of the proposed method, it is necessary to make such comparisons with larger numbers of participants with different hand dimensions. However, this will require the development of a representative hand model by warping the present hand model based on a statistical database of hand dimensions, and a method to generate hand models of participants by deforming the representative hand model. These should also be addressed in future research.

## Data Availability

The datasets used and/or analysed during the current study available from the corresponding author on reasonable request.
